# USA300 methicillin-resistant *Staphylococcus aureus* in Stockholm, Sweden, from 2008 to 2016

**DOI:** 10.1371/journal.pone.0205761

**Published:** 2018-11-07

**Authors:** Josefine Enström, Inga Fröding, Christian G. Giske, Karolina Ininbergs, Xiangning Bai, Gustaf Sandh, Ulla-Britt Tollström, Måns Ullberg, Hong Fang

**Affiliations:** 1 Department of Clinical Microbiology, Karolinska University Hospital, Stockholm, Sweden; 2 Department of Laboratory medicine, Karolinska Institutet, Stockholm, Sweden; 3 Communicable Disease Control in Stockholm County, Stockholm, Sweden; Rockefeller University, UNITED STATES

## Abstract

Methicillin-resistant *Staphylococcus aureus* (MRSA) USA300 isolates have been recognized globally, not only in community but also in healthcare settings. USA300 isolates were initially resistant only to methicillin, but resistance to non-β-lactams has emerged with time. To evaluate the prevalence and antimicrobial susceptibility of USA300 isolates in Stockholm, we conducted a nine-year retrospective study. Of 5359 consecutive MRSA cases in Stockholm, isolates from 285 cases were USA300 strains according to the pulsed-field gel electrophoresis pattern. Of these cases, repeated isolates with altered antibiotic resistance patterns were observed in six individuals. Therefore, antimicrobial susceptibility testing was performed on totally 291 isolates. To study the phylogenetic relatedness of isolates in transmission events and genomic resistance traits, 35 isolates were further studied by whole genome sequencing (WGS). The incidence of MRSA was increased from 17.6 per 100,000 inhabitants in 2008 to 37.3 per 100,000 inhabitants in 2016, while the proportion of USA300 cases declined from 6.6% in 2008 to 2.6% in 2016. Among the USA300 isolates, 73.5% were community-associated, 21.3% healthcare-associated, and 5.2% had unknown acquisition. The highest resistance rate among non-β-lactams was found in erythromycin (86%), followed by fluoroquinolones (68–69%). 57% of the isolates were resistant to both erythromycin and fluoroquinolone. Simultaneous resistance to four non-β-lactam antibiotic classes was found in six isolates. Four isolates were susceptible to all non-β-lactam antibiotics. Ceftaroline, daptomycin, linezolid, mupirocin, rifampicin, teicoplanin, telavancin, trimethoprim-sulfamethoxazole and vancomycin retained full activity in the study. WGS analysis indicated that isolates from an outbreak were phylogenetically closely related. In conclusion, USA300 MRSA isolates in Stockholm have neither been limited to the community setting, nor remained susceptible to non-β-lactam agents. WGS is becoming a useful tool in tracing transmission events. The results herein provide the most up-to-date and comprehensive information regarding status of USA300 strains in this geographic area.

## Introduction

Methicillin-resistant *Staphylococcus aureus* (MRSA) USA300 emerged first as community-associated MRSA in the USA in the late 1990s [1, 2]. USA300 strains spread rapidly across the USA, and presently predominate not only in community, but also in healthcare settings. Subsequently, USA300 isolates have been recognized globally. It is likely that USA300 strains are now endemic pathogens worldwide, and not simply imported strains [2]. The first USA300 MRSA case in Stockholm was observed in January 2004 in a patient who had acquired MRSA abroad. In the following years the USA300 strains were reported as community-associated [3]. As occurred in other countries, these strains are now emerging as healthcare-associated MRSA (HA-MRSA) in Stockholm.

USA300 isolates were initially resistant only to methicillin, but resistance to macrolides and fluoroquinolones has emerged with time. Plasmid-mediated clindamycin resistance, high-level mupirocin resistance, and tetracycline resistance have been reported among them [4]. Several USA300 isolates have developed non-susceptibility to vancomycin, and, in some cases, to daptomycin, in addition to occasional resistance to gentamicin and trimethoprim-sulfamethoxazole [2].

This laboratory-based surveillance study aimed to investigate the prevalence and antimicrobial susceptibility of USA300 isolates in Stockholm, Sweden during 2008–2016, and illustrate the phylogenetic relatedness of certain isolates in transmission events by whole genome sequencing (WGS).

## Materials and methods

### Case collection

MRSA has been notifiable according to the Swedish Communicable Disease Act since the year 2000 [5]. The Department of Clinical Microbiology at Karolinska University Hospital (Stockholm, Sweden) is a referral laboratory of MRSA isolates from all microbiology laboratories in Stockholm County, and has performed epidemiological typing for all MRSA cases detected in this region since 2008. The total population in the area was 1,981,263 and 2,269,060 reported on 31 December 2008 and 2016, respectively.

### Bacterial isolates

Of 5359 consecutive MRSA cases in Stockholm during 2008–2016, isolates from 285 cases were USA300 strains according to the pulsed-field gel electrophoresis pattern (PFGE). Of these cases, repeated isolates with altered antibiotic resistance patterns were observed in six individuals. Therefore, in total 291 isolates were further investigated in the present study. The PFGE USA300 type was defined by comparison with the pattern of HARMONY strain SE03-5 (multilocus sequence type ST8, *spa* t008, SCC*mec* IV, *pvl-*positive) according to the International Union of Microbiology Societies’European Staphylococcal Typing Network [6, 7], and USA300 reference strain *S*. *aureus* ATCC BAA-1717.

### Antimicrobial susceptibility testing

The following 19 antimicrobial agents were tested in the study: cefoxitin (FOX), ceftaroline (CPT), clindamycin (CLI), daptomycin (DAP), erythromycin (ERY), fusidic acid (FUS), gentamicin (GEN), levofloxacin (LVX), linezolid (LZD), moxifloxacin (MXF), mupirocin (MUP), norfloxacin (NOR), rifampicin (RIF), teicoplanin (TEI), telavancin (TLA), tetracycline (TET), tobramycin (TOB), trimethoprim-sulfamethoxazole (SXT) and vancomycin (VAN). The minimum inhibitory concentrations (MICs) of the antimicrobial agents for each isolate were determined using the broth microdilution method following EUCAST guidelines [8], in 96-well EUSTAPF plates (Sensititre, Thermo Scientific, UK). *S*. *aureus* ATCC 29213 was used as quality control strain.

For isolates with gentamicin or tobramycin MICs at > = 8 μg/ml by the Sensititre system, Etest (bioMérieux SA, France) was performed to further determine MICs higher than 8 μg/ml.

Strains were defined as being multidrug-resistant (MDR) when resistance was observed in two or more antimicrobial agents of non-β-lactam categories.

### Whole genome sequencing

WGS was performed on 35 isolates, including six isolates concomitant resistant to four non-β-lactam antibiotics, four multi-susceptible isolates, 24 isolates with known epidemiological relatedness and the USA300 reference strain *S*. *aureus* ATCC BAA-1717. Bacteria were pretreated by lysozyme and lysostaphin, then followed by extraction of genomic DNA with the EZ1 Advanced XL system (QIAGEN). Sequencing was done on Illumina HiSeq 2500 in SciLifeLab (Stockholm, Sweden), generating paired-end sequences in ≥30x coverage.

The antibiotic resistance genes, genetic mutations, virulence factors and SCC*mec* types were identified from whole genome sequences by using an online platform 1928 Diagnostics version 1.0 (https://1928diagnostics.com/).

Twenty-four isolates with known epidemiological relatedness, together with the USA300 reference strain *S*. *aureus* ATCC BAA-1717, were further investigated by core genome MLST (cgMLST, 1928 Diagnostics), single nucleotide polymorphisms (SNPs) and *k*-mer analysis (CLC Genomic Workbench, Microbial Genomic Module, QIAGEN). The cgMLST scheme is based on 1750 core genes. The minimum criteria for a qualified cgMLST analysis is that more than 95% of the core genes are identified. A *k*-mer tree was constructed, based on *k*-mers by using Feature Frequency Profile (FFP) method.

## Results

### Prevalence of MRSA cases

**Fig 1:**
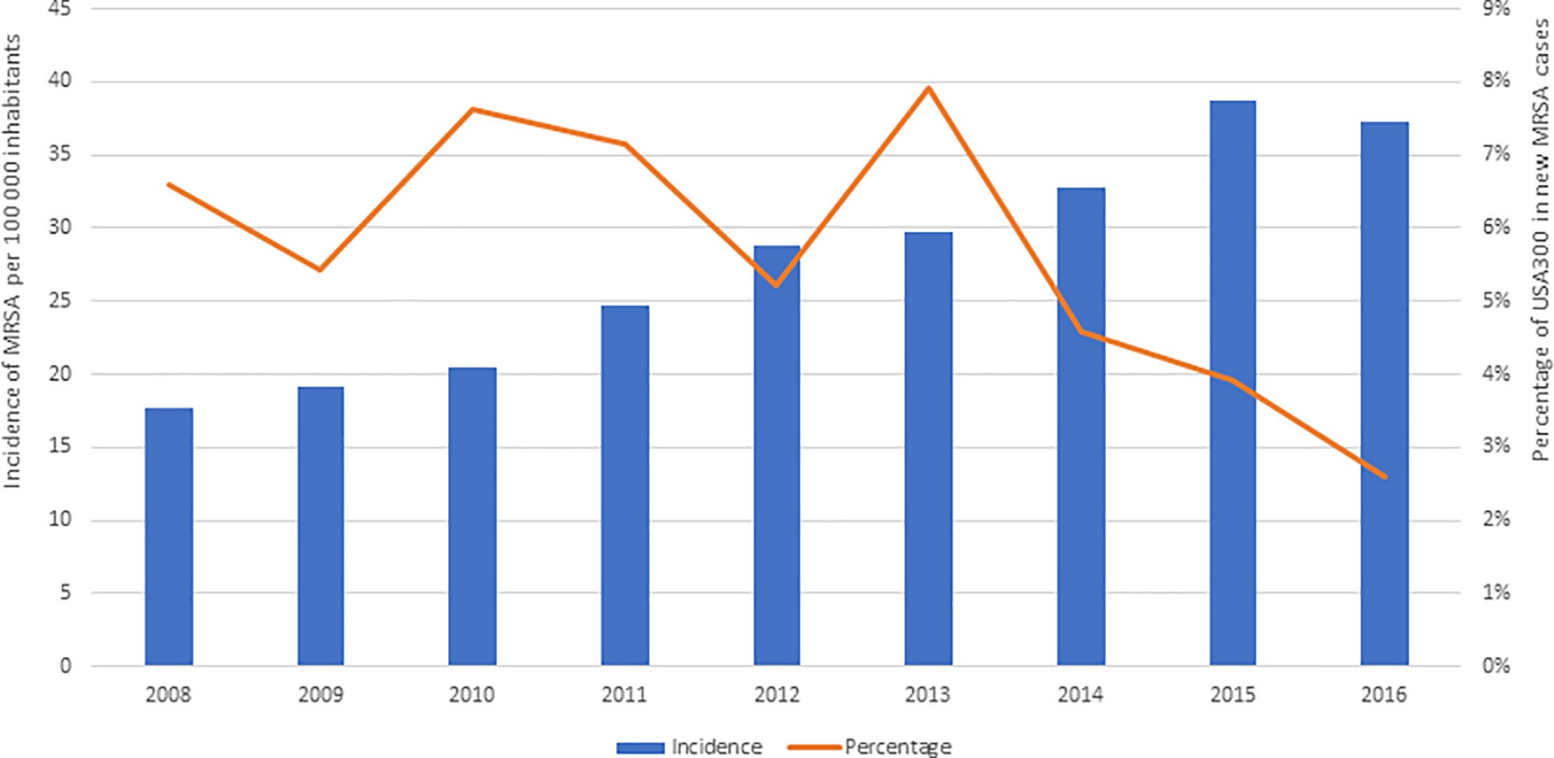
MRSA incidence. The incidence of MRSA and the percentage of USA300 out of new MRSA cases per year from 2008 through 2016 in Stockholm, Sweden.

Totally 5359 MRSA cases were documented in Stockholm during 2008–2016. The incidence of MRSA was increased from 17.6 per 100,000 inhabitants in 2008 to 37.3 per 100,000 inhabitants in 2016, while the proportion of USA300 cases declined from 6.6% in 2008 to 2.6% in 2016 with a peak value at 7.9% in 2013 (Fig 1, S1 Table).

Among the USA300 isolates, 73.5% were community-associated (CA-MRSA), 21.3% healthcare-associated (HA-MRSA), and 5.2% had unknown acquisition. Patient data were reviewed to distinguish CA-MRSA strains from HA-MRSA strains by epidemiological criteria. Strains were defined as CA-MRSA if MRSA was detected in an outpatient setting or by a positive culture for MRSA within 48 h after hospital admission. Moreover, CA-MRSA carriers should not have had 1) a medical history of MRSA colonization or infection; 2) a history of hospitalization, admission to a nursing home or hospice, dialysis, or surgery during the previous year; or 3) permanent indwelling percutaneous catheters or medical devices. Strains that did not meet the definition of CA-MRSA were classified as HA-MRSA [9, 10]. Strains lacking epidemiological information were classified as unknown acquisition. The 60 HA-MRSA cases consisted of 17 cases transmitted in Sweden and 43 acquired abroad. The abroad-acquired cases came from Bangladesh, Chile, Cuba, India, Indonesia, Italy, Jamaica, Japan, Mongolia, Pakistan, Philippines, Somalia, Spain, Switzerland, Syria, Taiwan, Thailand, Turkey, United Arab Emirates and the United States. Among the 43 abroad-acquired cases, 24 patients had been hospitalized and 19 cases were outpatients before they were diagnosed MRSA-positive in Sweden.[10]

### Antimicrobial susceptibility

Antimicrobial susceptibility testing was performed on 291 USA300 isolates detected in Stockholm from 2008 to 2016. All 291 isolates were susceptible to ceftaroline, daptomycin, linezolid, mupirocin, rifampicin, teicoplanin, telavancin, trimethoprim-sulfamethoxazole and vancomycin. The overall antimicrobial susceptibility profiles to the remaining antimicrobial agents are shown in Table 1.

**Table 1:** Resistance profiles of the 291 USA300 isolates in Stockholm, Sweden during 2008–2016.

	CLI	ERY	FOX	FUS	GEN	LVX	MXF	NOR	TET	TOB
**MIC range (μg/ml)**	< = 0.12 - >1	0.5 - >4	8 - >8	< = 0.5 - >4	< = 0.25–64	< = 0.5 - >4	< = 0.25 - >2	< = 4 - >16	< = 0.5 - >4	< = 0.25 - >8
**MIC**_**50**_ **(**μ**g/ml)**	< = 0.12	>4	>8	< = 0.5	< = 0.25	>4	2	>16	1	< = 0.25
**MIC**_**90**_ **(**μ**g/ml)**	< = 0.12	>4	>8	< = 0.5	< = 0.25	>4	>2	>16	1	0.5
**Resistance (%)**	2.4/2.7^#^	86	100	1	3	68.4	68.7	(69.8)*	9	3

* Breakpoint is not available. If R>4 mg/L, resistance rate was indicated in the parenthesis.

^#^ Resistant isolates/including inducible clindamycin resistance

CLI: clindamycin; ERY: erythromycin; FOX: cefoxitin; FUS: fusidic acid; GEN: gentamicin; LVX: levofloxacin; MXF: moxifloxacin; NOR: norfloxacin; TET: tetracycline; TOB: tobramycin.

Four isolates were susceptible to all non-β-lactam antibiotics tested in the study. Multidrug-resistant phenotypes, resistant to two or more antimicrobial agents of non-β-lactam categories, were detected in 176 isolates (61%). Fifty-seven percent of all isolates were resistant to both erythromycin and fluoroquinolone (FLQ). The most resistant patterns observed were ERY-FLQ-CLI-TET and ERY-FLQ-FUS-AMG. Resistance patterns and year of isolation are outlined in Table 2.

**Table 2:** Distribution of resistance patterns in 176 multidrug-resistant USA 300 isolates from Stockholm, Sweden during 2008–2016.

Multidrug-resistance pattern	2008 n (%)	2009 n (%)	2010 n (%)	2011 n (%)	2012 n (%)	2013 n (%)	2014 n (%)	2015 n (%)	2016 n (%)	Total n	Percent of all isolates
Total (%)	14 (60.9)	18 (85.7)	17 (53.1)	24 (64.9)	20 (62.5)	24 (47.1)	19 (55.9)	22 (62.9)	18 (69.2)	176	60.5%
Four-drug resistance	0	1	0	1	0	2	1	1	0	6	2.1%
ERY-LVX/MXF- CLI-TET	0	1	0	1	0	1	1	1	0	5	
ERY-LVX/MXF-FUS-GEN/TOB	0	0	0	0	0	1	0	0	0	1	
Three-drug resistance	1	2	2	4	3	3	0	2	2	19	6.5%
ERY-LVX/MXF-TET	1	2	1	2	0	3	0	2	1	12	
ERY-LVX/MXF-GEN/TOB	0	0	0	1	3	0	0	0	1	5	
ERY-LVX/MXF-CLI	0	0	0	1	0	0	0	0	0	1	
ERY-CLI-TET	0	0	1	0	0	0	0	0	0	1	
Two-drug resistance	13	15	15	19	17	19	18	19	16	151	51.9%
ERY-LVX/MXF	13	14	14	17	16	19	18	19	12	142	
LVX/MXF-GEN/TOB	0	0	0	2	1	0	0	0	0	3	
LVX/MXF-TET	0	0	0	0	0	0	0	0	2	2	
ERY-CLI	0	0	0	0	0	0	0	0	1	1	
ERY-TET	0	1	0	0	0	0	0	0	0	1	
FUS-GEN/TOB	0	0	0	0	0	0	0	0	1	1	
MXF-TET	0	0	1	0	0	0	0	0	0	1	

**Fig 2:**
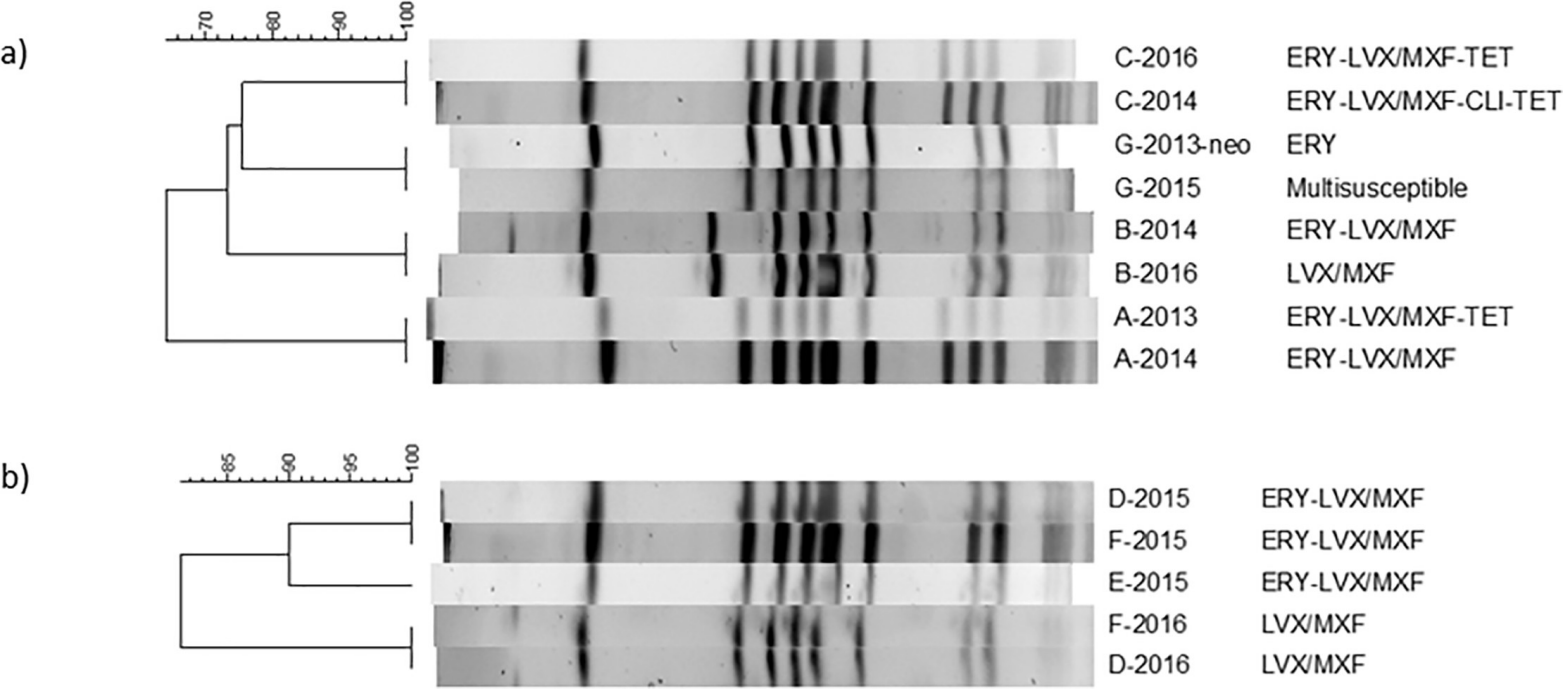
PFGE and resistance patterns. a) PFGE and resistance patterns of eight isolates, named as “case name-isolated year”, from cases A, B, C and G; b) PFGE and resistance patterns of isolates from cases D-F in a family.

Among the six cases from which subsequent isolates had altered resistance patterns, the second isolates lost resistance against tetracycline in case A, erythromycin in cases B, D, F and G, or clindamycin in case C (Fig 2).

### Genomic resistance traits, virulence factors and SCC*mec* types

The antimicrobial resistance genes and genetic mutations found in the multidrug-resistant isolates are presented in Table 3. The genomic resistance traits and the phenotypical resistance profiles were in accordance among the investigated isolates. Resistance genes *erm*C, *msr*A and *lnu*A were identified in the isolates resistant to erythromycin and klindamycin. S84L mutation in *gyr*A, S80Y, S80F or E84K mutations in *grl*A genes were observed in the quinolone-resistant isolates. *tet*M or *tet*K genes were detected in the isolates resistant to tetracyclines. Mutation (L461K) in *fus*A gene and the presence of *aac6-aph2* were responsible for the resistance to fusidic acid and aminoglycosides, respectively.

**Table 3:** Genomic resistance traits detected in the four-drug-resistant USA300 isolates (n = 6) by the online platform 1928 Diagnostics.

Resistance pattern	No of isolates	Macrolides-Lincosamides	Quinolones	Tetracyclines	Fusidic acid	Aminoglycosides
ERY-LVX/MXF- CLI-TET	1	*ermC*	*gyrA* (S84L) *grlA* (S80Y) + *gyrA* (S84L)	*tetM*	-	
	1	*ermC*	*grlA* (S80Y) + *gyrA* (S84L)	*tetK*	-	
	3	*msrA* *ermC*	*grlA* (S80Y) + *gyrA* (S84L)	*tetK*	-	
ERY-LVX/MXF-FUS-GEN/TOB	1	*msrA*, *lnuA*	*grlA* (S80F) *gyrA* (S84L) *grlA* (S80F) + *gyrA* (S84L) *grlA* (E84K) + *gyrA* (S84L) *grlA* (S80F + E84K) + *gyrA* (S84L)	-	*fusA* (L461K)	*aac6*-*aph2*

Of the four multi-susceptible isolates, *msr*A was present in one isolate which was phenotypically susceptible to erythromycin (MIC: 0.5 μg/ml). No other resistance traits for non-β-lactams were detected among them.

Genes encoding PVL toxin were detected in all WGS-investigated isolates except for the isolates from case A (isolates: A-2013 and A-2014). SCC*mec* IV was present in all isolates investigated.

The WGS raw data are available at National Center for Biotechnology Information (NCBI, Bethesda MD, USA) Sequence Read Archive (SRA) (S2 Table).

### Phylogenetic relatedness of the isolates in the transmission events

Nowadays, the ongoing evolution of sequencing technologies from Sanger sequencing to next-generation sequencing enables sequence analysis on a whole-genome level. WGS-based typing on gene-by-gene allelic profiling of core genome genes, named core genome MLST (cgMLST), single nucleotide polymorphisms (SNPs), or *k*-mer-based DNA sequence analysis in a species’ genome provides the discriminative power needed for outbreak investigation of clonal pathogens.

**Fig 3:**
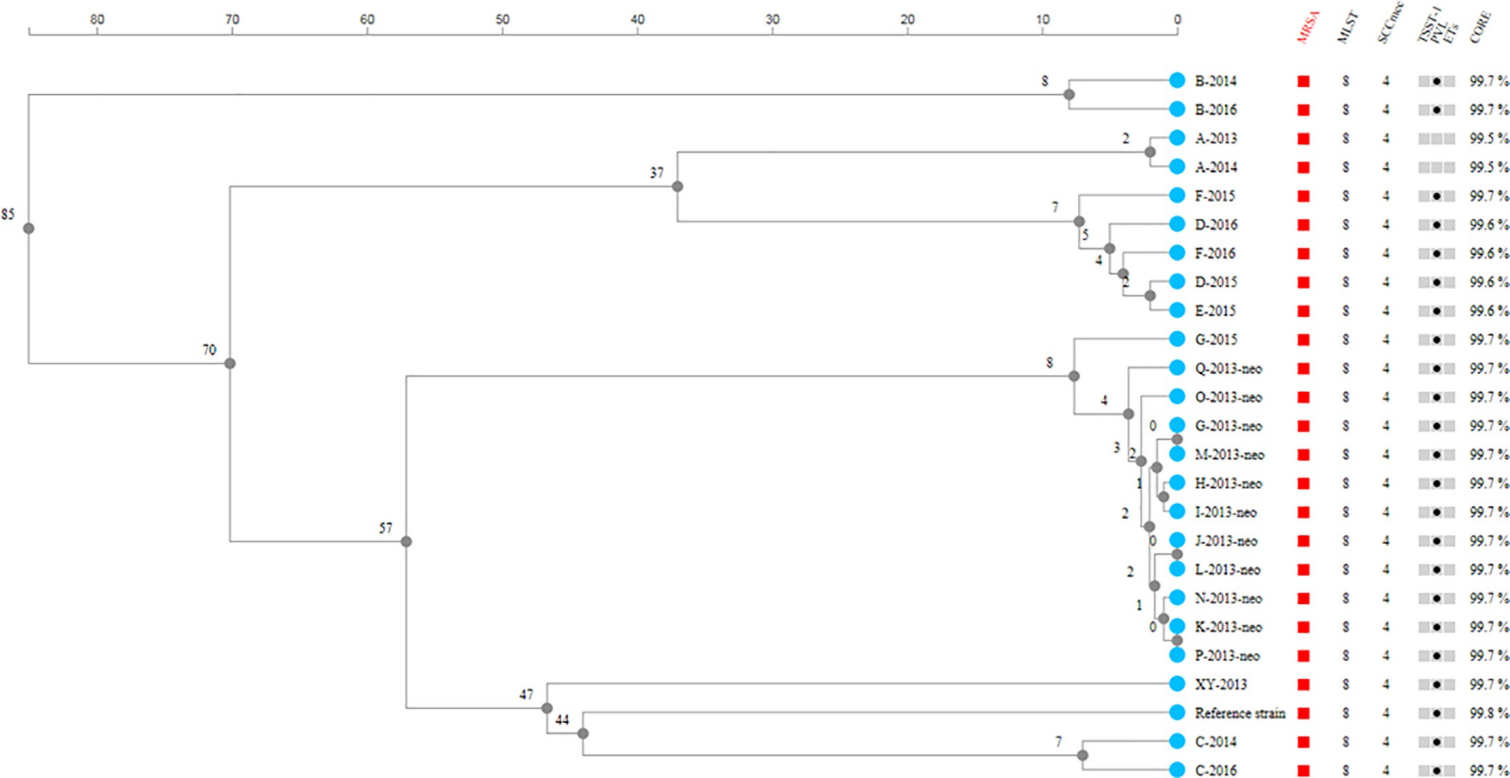
cgMLST phylogenomic tree. The cgMLST phylogeny of USA300 isolates in the transmission events together with USA300 reference strain *S*. *aureus* ATCC BAA-1717. The scale shows the number of loci with different alleles.

**Fig 4:**
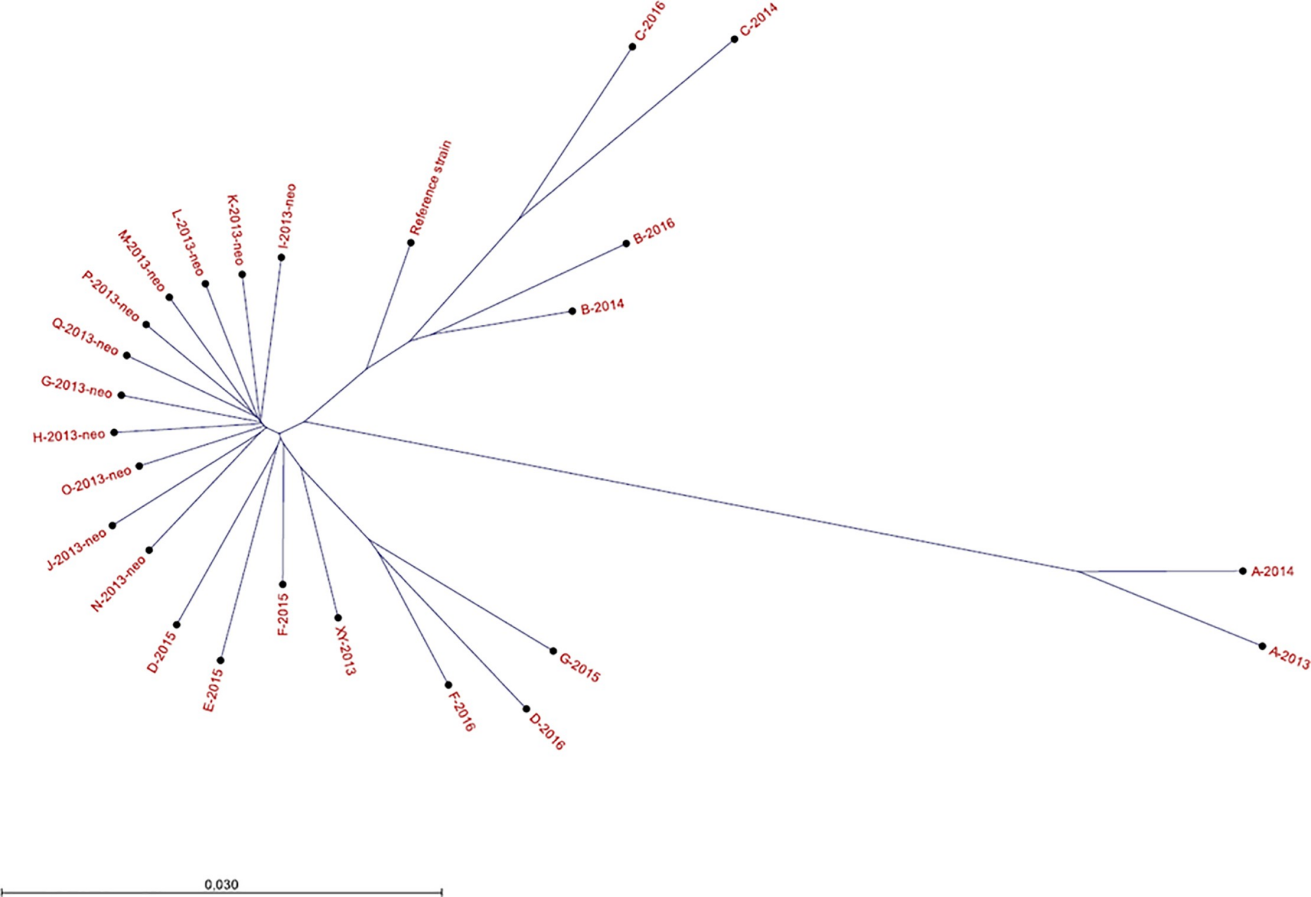
*K*-mer phylogenomic tree. The *k*-mer tree of USA300 isolates in the transmission events (Family transmission: D, E, F; Outbreak: G-Q; Not epidemiologically related: XY) and recurrent isolates (A, B, C, D, F, G) together with USA300 reference strain *S*. *aureus* ATCC BAA-1717. The scale bar refers to the branch lengths within the tree. The branch length of each isolate to its nearest node is between 0 (exactly same *k*-mer distribution) and 1 (completely different *k*-mer distribution).

Both the cgMLST phylogeny (Fig 3) and *k*-mer tree (Fig 4) showed that 11 isolates (G-2013-neo–Q-2013-neo) recovered from an outbreak in the department of neonatal in the fall of 2013 clustered tightly, and they were distinct from an isolate (XY-2013) recovered from another ward in the same period, thus implying close phylogenetic relatedness of outbreak isolates. The isolates in the outbreak differed in up to three SNPs and up to four core-genes, while the non-outbreak isolate XY-2013 showed difference in 141–144 SNPs and 57 core-genes from the outbreak isolates.

Isolates with altered resistance patterns from the same patients (A, B, C), also showed tendency to cluster closely. Isolates of the same patient over 1–2 years had 13–40 SNPs (Table 4). Regarding case G, the first isolate (G-2013-neo) was closer to the outbreak isolates of other patients (0–2 SNPs) than the subsequent isolate of herself in 2015 (G-2015, 29 SNPs). Notably, isolates from members (D, E, and F) of the same family were grouped together. The family-transmitted isolates showed 3–13 SNPs in the transmission event, and up to 41 SNPs one year after (Table 4). In the cgMLST analysis, the five isolates of the same family differed in up to seven core-genes (Fig 3).

**Table 4:** SNP matrix of the whole-genome sequences from the cases with repeated isolation of MRSA and the family-transmitted cases.

Samples*	A-2013	A-2014	B-2014	B-2016	C-2014	C-2016	D-2015	D-2016	E-2015	F-2015	F-2016	G-2013-neo	G-2015
**A-2013**	0	37	354	372	304	309	232	263	235	240	262	322	351
**A-2014**	37	0	323	341	336	341	201	232	204	209	231	291	320
**B-2014**	354	323	0	31	209	216	206	237	209	214	236	185	214
**B-2016**	372	341	31	0	229	234	224	237	227	232	236	203	214
**C-2014**	304	336	209	229	0	13	219	250	222	227	249	198	227
**C-2016**	309	341	216	234	13	0	224	255	227	232	254	203	232
**D-2015**	232	201	206	224	219	224	0	33	5	10	32	174	203
**D-2016**	263	232	237	237	250	255	33	0	28	41	3	205	190
**E-2015**	235	204	209	227	222	227	5	28	0	13	27	177	206
**F-2015**	240	209	214	232	227	232	10	41	13	0	40	182	211
**F-2016**	262	231	236	236	249	254	32	3	27	40	0	204	189
**G-2013-neo**	322	291	185	203	198	203	174	205	177	182	204	0	29
**G-2015**	351	320	214	214	227	232	203	190	206	211	189	29	0

* Samples were named as “case name—isolated year”. Cases D, E and F were members of the same family.

## Discussion

This study systematically investigated the regional epidemiology and susceptibility patterns of USA300 MRSA isolates identified, on a routine basis, in Stockholm County.

Nordic countries, including Sweden, are known for a relatively low prevalence and incidence of MRSA. Besides the low prevalence of MRSA, the effective infection control is also reflected in the high genetic diversity and absence of predominant genotypes among the isolates in the area [3, 11, 12]. Our previous findings demonstrated that no PFGE type accounted for >10% of all MRSA isolates and that USA300 was the second most common PFGE type in Stockholm in 2014 [11].

In the present study, it was observed that the percentage of USA300 varied between 5.2% and 7.9% during 2008–2013 and reached a maximum in 2013. A similar scenario was depicted in a study in Norway [13]. However, it was found that USA300 has increased in incidence in parallel with the total MRSA incidence from 2003 to 2011 in Norway, which was not observed in our study. After 2013, we observed a decreasing trend in the incidence of detected USA300 cases (Fig 1). The changed incidence of USA300 in the latest years might indicate an even more diverse heterogeneity of MRSA in Stockholm, but the replacement by another clone could not be excluded without further investigation. During the whole study period (2008–2016), USA300 accounted for 5.3% of all cases in Stockholm, which was comparable with a study (5.6%) in Zurich, Switzerland [14]. In 2013, an outbreak with USA300 MRSA took place in neonatal departments in Stockholm, in which six neonates, three mothers and two healthcare-workers were involved.

The epidemiology of MRSA is changing, with USA300 emerging initially in the community and later in healthcare settings [1]. A Norwegian study demonstrated that most USA300 isolates (76.4%) in the study area were found in the community, but isolates were also detected in healthcare settings [13]. Likewise, in Stockholm 73.5% of the USA300 isolates were community-associated, of which 14% were family transmitted.

USA300 MRSA isolates frequently remain susceptible to non-β-lactam agents, but trends of increasing evolution of antibiotic resistance have been observed [15, 16]. In an international survey on USA300 recovered in 2009 and 2010 [15], the lowest susceptibility was observed to erythromycin in all regions, and the highest susceptibility was reported to linezolid, rifampicin, trimethoprim-sulfamethoxazole and vancomycin. A study from USA reported 92% and 53% non-susceptibility to erythromycin and levofloxacin, while nearly full susceptibility to ceftaroline, daptomycin, linezolid, trimethoprim-sulfamethoxazole and vancomycin was observed among USA300 isolates from 43 US centers in 2011. Similar to these reports, the highest resistance rate among non-β-lactams was also found to erythromycin in Stockholm, followed by fluoroquinolone (Table 2).

In the present study, resistance to aminoglycosides were detected in ten isolates (3%), which were resistant to both gentamicin and tobramycin with MICs at 16–64 μg/ml for gentamicin and 8–64 μg/ml for tobramycin. Although resistance to clindamycin (2%) or fusidic acid (1%) could be demonstrated among the Stockholm USA300 isolates, clindamycin and fusidic acid retained activity against most isolates investigated in the present study.

The susceptibility patterns observed in USA300 isolates in Stockholm are generally in accordance with reports from the USA, Brazil and Canada, but different from those European isolates [1, 15]. European countries in the international survey were represented by Denmark, France, Germany, Greece, Italy, Netherland, Poland and Switzerland [15]. Since the situation of antimicrobial resistance varies a lot in European countries [17], the variation in proportion of isolates from these countries could lead to a diverging total picture.

The most noticeable feature of the USA300 genome is its rapid diversification and acquisition of different mobile genetic elements, which contributes to its evolution and to emergence of new multidrug-resistant strains of this successful clone [18]. In the present study, the most resistant patterns observed were simultaneously resistant to four non-β-lactam antibiotic classes, represented by six isolates (Table 2).

In Stockholm, 57% of USA300 isolates featured concomitant resistance to erythromycin and fluoroquinolones, which was also the most frequent resistance pattern reported in Brazil, Canada and the USA [1, 15]. In a US study [1], resistance to erythromycin or levofloxacin was excluded from their definition for multidrug resistance, and therefore multidrug-resistant phenotypes were reported in 3% of USA300 isolates. In this way, eight isolates (2.7%) were categorized as multidrug-resistant among USA300 isolates from Stockholm.

Although there were few multidrug-resistant isolates (2.7%), the Stockholm USA300 isolates were no longer multi-susceptible to non-β-lactam agents. Only four isolates (1.4%) were found to be multi-susceptible (Table 2), which could be worrisome. Notably, there was a good concordance between the antimicrobial resistance phenotype and the presence of genomic resistance traits in this study.

According to the genomic phylogenetic analysis, it can be inferred from this study that isolates from an outbreak were phylogenetically closely related. Species-specific cutoffs for the putative outbreak isolates are not yet well-established in WGS-based typing. Our study showed that the outbreak isolates (USA300) differed in up to three SNPs and up to four core-genes, which were within the reported thresholds for *S*. *aureus* [19]. Isolates from same patients or the same family might evolve over time, but still cluster together (up to 41 SNPs and eight core-genes) in our study. Genomic variability (*i*.*e*. clock speed) in these actively growing isolates affects the ultimate strain typing issue of relatedness.

In conclusion, USA300 MRSA isolates have neither been limited to the community setting, nor remained susceptible to non-β-lactam agents in Stockholm. With its potential to become epidemic and increasingly resistant to additional antibiotic classes, USA300 MRSA should be well monitored when identified in an area. WGS is becoming a useful tool in tracing transmission events. Our results provide the most up-to-date and comprehensive information regarding status of USA300 strains in Stockholm, Sweden.

## Supporting information

S1 Table MRSA and USA300-type MRSA in Stockholm from 2008 to 2016. (DOCX)

S2 Table Accession numbers of WGS raw data at NCBI Sequence Read Archive. (DOCX)
