# Supplementary material for: USA300 methicillin-resistant Staphylococcus aureus in Stockholm, Sweden, from 2008 to 2016
Source: PLoS One. 2018 Nov 7;13(11):e0205761. doi: 10.1371/journal.pone.0205761 (PMC6221263; doi:10.1371/journal.pone.0205761)
Supplement: S1 Table — (DOCX) [file pone.0205761.s001.docx]

**S1 Table. MRSA and USA300-type MRSA in Stockholm from 2008 to 2016.**

| **Year** | **USA300 isolates in the study** | **USA300 cases** | **New MRSA cases per year** | **Population in Stockholm county** | **Incidence of MRSA per 100 000 inhabitants** | **Percentage of USA300 in new MRSA cases** |
| --- | --- | --- | --- | --- | --- | --- |
| **2008** | 23 | 23 | 349 | 1 981 263 | 17.62 | 6.6% |
| **2009** | 21 | 21 | 387 | 2 019 182 | 19.17 | 5.4% |
| **2010** | 32 | 32 | 420 | 2 054 343 | 20.44 | 7.6% |
| **2011** | 37 | 37 | 517 | 2 091 473 | 24.72 | 7.2% |
| **2012** | 32 | 32 | 612 | 2 127 006 | 28.77 | 5.2% |
| **2013** | 51 | 51 | 644 | 2 163 042 | 29.77 | 7.9% |
| **2014** | 34 | 33 | 719 | 2 198 044 | 32.71 | 4.6% |
| **2015** | 35 | 34 | 865 | 2 231 439 | 38.76 | 3.9% |
| **2016** | 26 | 22 | 846 | 2 269 060 | 37.28 | 2.6% |
| **Total** | 291 | 285 | 5359 |  |  | 5.3% |
