# Supplementary material for: USA300 methicillin-resistant Staphylococcus aureus in Stockholm, Sweden, from 2008 to 2016
Source: PLoS One. 2018 Nov 7;13(11):e0205761. doi: 10.1371/journal.pone.0205761 (PMC6221263; doi:10.1371/journal.pone.0205761)
Supplement: S2 Table — (DOCX) [file pone.0205761.s002.docx]

**S2 Table. Accession numbers of WGS raw data at NCBI Sequence Read Archive.**

| **Sample name** | **Accession nr at NCBI** |
| --- | --- |
| A-2013 | SAMN10054248 |
| A-2014 | SAMN10054249 |
| B-2014 | SAMN10054250 |
| B-2016 | SAMN10054251 |
| C-2014 | SAMN10054252 |
| C-2016 | SAMN10054253 |
| D-2015 | SAMN10054254 |
| D-2016 | SAMN10054255 |
| E-2015 | SAMN10054256 |
| F-2015 | SAMN10054257 |
| F-2016 | SAMN10054258 |
| G-2013-neo | SAMN10054259 |
| G-2015 | SAMN10054260 |
| H-2013-neo | SAMN10054261 |
| I-2013-neo | SAMN10054262 |
| J-2013-neo | SAMN10054263 |
| K-2013-neo | SAMN10054264 |
| L-2013-neo | SAMN10054265 |
| M-2013-neo | SAMN10054266 |
| N-2013-neo | SAMN10054267 |
| O-2013-neo | SAMN10054268 |
| P-2013-neo | SAMN10054269 |
| Q-2013-neo | SAMN10054270 |
| XY-2013 | SAMN10054271 |
| Reference strain | SAMN10054272 |
| Multi-resistant 1 | SAMN10054273 |
| Multi-resistant 2 | SAMN10054274 |
| Multi-resistant 3 | SAMN10054275 |
| Multi-resistant 4 | SAMN10054276 |
| Multi-resistant 5 | SAMN10054277 |
| Multi-resistant 6 | SAMN10054278 |
| Susceptible 1 | SAMN10054279 |
| Susceptible 2 | SAMN10054280 |
| Susceptible 3 | SAMN10054281 |
| Susceptible 4 | SAMN10054282 |
